# Relationship Between Break-Time Physical Activity, Age, and Sex in a Rural Primary Schools, Wales, UK

**DOI:** 10.2478/hukin-2014-0024

**Published:** 2014-04-09

**Authors:** Yolanda Escalante, Karianne Backx, Jose M. Saavedra

**Affiliations:** 1 Facultad de Ciencias del Deporte, AFIDES Research Group, Universidad de Extremadura, Caceres, Spain.; 2 Cardiff School of Sport, Cardiff Metropolitan University, Cardiff, United Kingdom.

**Keywords:** Childhood, exercise, health, sedentary lifestyle

## Abstract

The aim of this study was to examine the physical activity during the break-times of primary school children in rural areas, and its relationship with age and sex. 380 children (192 boys and 188 girls; age=9.5±1.1 years) participated in the study. Break-time physical activity in the morning and lunch breaks was measured by accelerometry. An ANOVA was used to determine differences by sex in each age group, together with the respective confidence intervals and effect sizes. The results showed that 8-year-olds performed more physical exercise than 11-year-olds during the two breaks (p=0.005). For the boys, the 8-year-olds did more physical activity than the 10-year-olds, while, for the girls, those aged 8 and 9 years did more PA than girls aged 11 years (p<0.001). The only difference between boys and girls was for the 10-year-olds (p=0.043), with the boys doing more physical activity. Teachers might find it useful to take these findings into account to design physical activity programmes aimed at increasing the playground physical activity of older children.

## Introduction

Physical activity (PA) and exercise provide major health benefits (Armstrong and Welsman, 1996). It is often used preventively in healthy people (Cooke et al., 2013) or as an intervention in people with conditions associated with physical inactivity such as obesity (Beavers et al., 2014) or diabetes (Backx et al., 2011). Despite the well-known benefits of physical activity, sedentary habits are becoming increasingly common (Varo et al., 2003). This has led various agencies to establish recommendations of minimal practices of PA. Thus, the WHO (2010) indicates that adults aged 18–64 years should do at least 150 minutes of moderate-intensity aerobic PA throughout the week, or at least 75 minutes of vigorous-intensity aerobic PA throughout the week, or an equivalent combination of moderate- and vigorous-intensity activity. Moreover, muscle-strengthening activities should be done involving major muscle groups on 2 or more days a week.

For children, the situation is similar. Sedentary lifestyles are reaching alarming levels in developed countries (Iannotti and Jing Wang, 2013). This, in association with unhealthy eating habits, sometimes leads to an increase in the prevalence of such diseases as obesity (Yngve et al., 2007) or even hypertension, a disorder considered until recently to be exclusive to adults (Barrios et al., 2013). Current guidelines recommend that children and adolescents (5–17 years) should engage in 60 minutes or more of daily PA, mainly aerobic and moderate or vigorous in intensity. Furthermore, it is also recommended that children participate in activities that strengthen the musculoskeletal system at least three times a week (WHO, 2010). However, despite these guidelines, only 27% of all girls and 40% of all boys follow these recommendations (WHO, 2010). Regarding the practice of PA as a function of the type of town or village the children live in, children of rural communities are more physically active than those of urban areas, while the latter use more screen time in their everyday lives (Salom et al., 2013). Also, the type of PA seems to be different. Children who live in rural areas spend more time in outdoor activities, but participate in less structured PA, than children living in urban areas (Donatiello et al., 2013). Furthermore, one has to bear in mind that children’s practice of PA depends strongly on the physical and social factors operating within their home environment (Maitland et al., 2013).

If the aim is to increase PA, it is necessary to know when it is possible for the child to do it, and what and where are their preferences. Children spend many hours in class at school, to which must be added their extracurricular activities. The school would thus seem to be an appropriate place for the promotion of PA (Dessing et al., 2013). In this regard, there are two key times at which to focus on the practice of PA: physical education classes and during break time. The former should be offered daily, with lessons engaging the children in moderate to vigorous PA for at least 50% of the class time (United States Department of Health and Human Services, 2000). Unfortunately, this is not always the case (Straton, 2000). Regarding the latter, the playground can constitute a good opportunity to increase PA levels. There have been studies indicating that, in general, boys do more PA than girls (Dessing et al., 2013), and that younger ages are more active than older (Escalante et al., 2011). The size of the playground also influences the amount of PA done (Escalante et al., 2012). Other studies have examined the effectiveness of different interventions in the school yard during break (Stratton, 2000; Stratton and Mullan, 2005; Verstraete et al., 2006; Ridgers et al., 2007a, b; Cardon et al., 2009; Ridgers et al., 2010; Huberty et al., 2011; Blaes et al., 2013). In this sense, a recent systematic review concluded that interventions based on playground markings, game equipment, or the combination of the two do not seem to increase the PA of preschoolers and schoolchildren during break, while interventions based on playground markings plus physical structures do, at least in the short to medium term (Escalante et al., 2013). However, most studies have been on urban (Stratton, 2000; Verstraete et al., 2006; Cardon et al., 2009; Huberty et al., 2011; Blaes et al., 2013.) or urban and rural populations (Ridgers et al., 2007a, b; Cardon et al., 2009; Ridgers et al., 2010; Huberty et al., 2011; Blaes et al., 2013; Escalante et al., 2011, 2012). Therefore, the aim of this study was to describe the PA during break of Primary School children in rural areas and its relationship with age and sex.

## Material and Methods

### Participants

Five schools in rural Wales were invited to participate in the study. Initially, 90% of those invited (the parents of 425 children) gave their written informed consent to take part. Of these, 45 were excluded from the data analysis. The reasons were non-completion of the general questionnaire (29 children), or problems with the accelerometer (16 children). The final sample thus comprised 380 children (9.5 ± 1.1 years, from 8 to 11 years).

### Measures

The participants’ body height and body mass were measured, and their break-time PA was determined by accelerometry, a method widely used within paediatric populations (Rowlands, 2001). The device used was a Caltrac accelerometer (Hemokinetics, Madison, WI, USA) programmed to operate in PA monitoring mode (Sallis et al., 1990). This uniaxial instrument has a piezoelectric bender that measures motion in the vertical plane. It integrates the absolute values of the acceleration forces and sums the motion counts. Measurements made with it have been demonstrated to be strongly correlated with those of a triaxial accelerometer (Eisenmann et al., 2004). The method is similar to that followed in other studies (Escalante et al., 2011; Escalante et al., 2012; Kimm et al., 2000; Sigmund et al., 2009; Saglam et al., 2010).

### Procedures

After the initial contact with the schools, and acceptance on the part of the head teachers, the children’s parents/guardians were asked to provide their written informed consent. This duly signed written consent form was a prerequisite for the child to be included in the study. The children’s body height and body mass were measured at the beginning of the day. About ten minutes before break-time, the researchers went into the classroom to distribute the accelerometers to the participants, who were seated at their desks. Each accelerometer was attached to the waistband of the child’s trousers or skirt before they went out to their playground. The accelerometer’s display was covered with black masking tape to prevent its measurements from being watched. The teachers and researchers monitored the break to ensure that the children’s PA did not differ from their usual activities. When they came back into class after the break, they put their accelerometers into a plastic collection bin. In all the schools, there were two 15-minute breaks, with the children going outside during both breaks. All data collection took place on sunny days. All age groups were in the playground at the same time, and 40 children were evaluated during each break period. The study was approved by the Commission of Ethics of Cardiff Metropolitan University, and complied with the principles of the Declaration of Helsinki.

### Statistical Analysis

The data analysis design was descriptive and cross-sectional, with age and sex as the independent variables, and the PA during the break as the dependent variable. The normality and homoscedasticity of the distributions was evaluated with the Kolmogorov-Smirnov and Levene tests, respectively. A one-way analysis of variance (ANOVA) was applied to test for intergroup equality of the means for the PA in the two break-times and the sum of the two. Scheffé’s post-hoc method was applied to account for multiple comparisons between age and sex groups. A *p*-value of <0.05 was taken as statistically significant. The confidence intervals and effect sizes of the differences were calculated (Cohen, 1988). All calculations were performed using the Statistical Package for Social Sciences (SPSS for Windows, version 19.0).

## Results

Table 1 presents the characteristics of the sample. All variables met the criteria of normality (0.250≤*z*≤0.963; *p*>0.05) and homoscedasticity (0.050≤*F*≤1.377, *p*>0.05).

Table 2 shows the PA results for the morning break and the lunch break, and their sum. For these two sets of data together, the 8-year-old children did more PA than those aged 11 years (*p*=0.005). The 8-year-old boys did more PA than those aged 10 years, while the 8- and 9-year-old girls did more activity than those aged 11 (*p*<0.001). Between the sexes, there were only differences between the 10-year-old boys and girls (*p*=0.043), with the former being more physically active.

Making this same analysis, but distinguishing between morning break and lunch break, we only observed differences in the latter. In terms of ages, these differences were between boys of 8 and 9 years (*p*=0.004), and between girls of 8 and 10 years (*p*=0.009). In terms of sex, there were only differences between the 10-year old boys and girls (*p*=0.036).

## Discussion

We have described a study of the PA performed by primary school children in rural primary schools in Wales during their school break times. The older children (11 years) performed less PA in the school yard during break time than the younger children (8 and 9 years). However, these differences were only present for the lunch break and for the whole break time (morning break plus lunch break). There were only differences with regard to sex for the 10-year-old children, with the boys being more active than the girls. Teachers might find it useful to take these findings into account to design proposals aimed at increasing the playground physical activity of older children.

The results showed 8-year-old boys to perform more PA during break (morning break plus lunch break) than those of 10 years in age (*p*=0.001), and that 8- and 9-year-old girls performed more PA than girls of 11 years in age (*p*<0.001). These findings contrast with a study conducted in Spain which only found differences in PA when the ages were more distant, i.e. 6 vs. 11 years old in girls and children (boys and girls) (Escalante et al., 2011). It should be noted, however, that the present study carried out in rural Welsh schools found levels of playground PA well above those of the Spanish schoolchildren. The reasons may be that playground PA is usually unstructured (Verstraete et al., 2006) and that this type of activity is predominant in rural areas (Donatiello et al., 2013). Also, PA during breaks is usually in short spurts of high intensity followed by rest or PA of less intensity (Lopes et al., 2006), the two short break periods in the Welsh schools accommodate this type of PA. The results are however consistent with other studies indicating that, after the age of 9, there is a reduction in daily PA in boys (Kimm et al., 2000; Stellino et al., 2010). Also, the lack of any differences in the morning break suggests that it is in the lunch break in which there is a greater possibility of intervention (Verstraete et al., 2006).

Regarding the differences by sex, these only existed for the 10-year-old children, with the boys being more active in the sum of the two break periods (p=0.043) and in the lunch break (p=0.036). These results are to be contrasted and compared with those of a previous study (Escalante et al., 2011) which found boys to perform a greater overall volume of accumulated PA than girls at the ages of 9, 10, and 11 years. Some studies have reported that maturity differences between the sexes (girls maturing earlier than boys) may be one reason why studies consistently find females to be less active than males of the same chronological age (Sherar et al., 2007). Intervention studies have found girls to present greater increases in light (O’Dwyer et al., 2013) and moderate-to-vigorous PA (Nettlefold et al., 2011). Therefore, other studies point to the opposite direction – that boys engage in more moderate-to-vigorous PA and vigorous PA during a morning break and a lunch break than girls (Ridgers et al., 2010). The differences by sex in the PA during a break are thus unclear (Nettlefold et al., 2011). This may be because the activities in which the boys and the girls are participating are themselves different (MacDonald et al., 2005). For example boys often participate in team activities such as football or rugby, i.e. activities of moderate to high intensity, which often contain a competitive element, whereas girls generally participate in activities of lower intensity such as talking, walking, or skipping (Beighle et al., 2005). Furthermore, the type of PA might be affected by physical and social environmental factors (Maitland et al., 2013).

The present study has two limitations. Firstly, wearing the accelerometers could have affected the children’s PA in the playground. Their teachers watched them during the measurement times, however, and told us that the children’s behaviour was no different from normal. Secondly, the PA was measured during the two break-times of just a single day. The sample size and the teachers’ observations that they had noticed no unusual activity may reduce the relevance of this limitation.

In conclusion, this study examined the PA performed by primary school children in rural contexts in Wales during their school break times as a function of age and sex. The main conclusions to be drawn are: (i) the older children (11 years in age) did less playground PA than the younger children (8 and 9 years); and (ii) only in the case of the 10-year-olds were the girls less active than the boys. Teachers might find it useful to take these findings into account to design proposals aimed at increasing the playground PA of older children. Nevertheless, in addition to pursuing the goal of increased PA, break time could also be used to improve basic physical skills by offering specific exercise programs (Yasumitsu and Nogawa, 2013).

## Figures and Tables

**Table 1:** Characteristics of sample. Data are mean ± standard deviation.

Characteristics	All children (n =380)	Boys (n =192)	Girls (n =188)	*p-*value
Body Height (m)	1.37 ± 0.89	1.37 ± 0.86	1.37 ± 0.93	0.535
Body Mass (kg)	36.21 ± 11.03	35.30 ± 10.04	37.05 ± 11.90	0.124
BMI (kg/m^2^)	18.33 ± 3.96	18.52 ± 3.61	19.44 ± 4.23	0.022

**Table 2:** Break-time physical activity (motion counts) of children according to age and sex. The ANOVA p-value (difference by sex and age), confidence interval, effect size, and Scheffé post-hoc p-value. Data are mean ± standard deviation.

Variable	8 years (a) (n=91)	9 years (b) (n=91)	10 years (c)(n=90)	11 years (d) (n=108)	*p*	Scheffé post- hoc
Break-time PA total (motion count)						
All children (n=380)	43.3 ± 22.5	43.0 ± 18.0	42.6 ± 24.5	39.9 ± 26.9	0.005	a<d
Boys (n=192)	44.3 ± 24.0	42.5 ± 14.0	46.9 ± 17.1	41.5 ± 25.9	0.001	c>d
Girls (n=188)	42.4 ± 21.0	43.5 ± 21.5	38.1 ± 20.8	37.9 ± 28.1	<0.001	a,b>d
Upper confidence interval	11.28	6.47	18.84	13.95		
Lower confidence interval	−7.53	−3.52	−1.46	−6.69		
*p*-value, boys vs girls	0.693	0.775	0.043	0.488		
Effect size	0.08	−0.06	0.46	0.13		

Morning break PA (motion count)						
All children (n=380)	23.4 ± 12.0	23.4 ± 9.7	21.5 ± 11.7	20.5 ± 12.3	0.060	
Boys (n=192)	24.2 ± 12.8	23.2 ± 8.1	23.5 ± 12.3	21.8 ± 12.0	0.223	
Girls (n=188)	22.6 ± 11.2	23.6 ± 11.0	19.4 ± 10.6	18.9 ± 12.5	0.131	
Upper confidence interval	6.61	3.71	9.05	7.60		
Lower confidence interval	−3.39	−4.45	−0.61	−1.78		
*p*-value, boys vs girls	0.523	0.857	0.118	0.222		
Effect size	0.13	−0.04	0.36	0.24		

Lunch break PA (motion count)						
All children (n=380)	19.9 ± 12.7	19.6 ± 10.1	21.0 ± 16.0	19.9 ± 14.1	0.854	b<c
Boys (n=192)	20.0 ± 14.2	19.3 ± 8.5	23.3 ± 19.5	19.7 ± 20.5	0.004	a>c
Girls (n=188)	19.7 ± 11.2	19.7 ± 11.4	17.8 ± 12.7	19.0 ± 17.0	0.009	
Upper confidence interval	5.59	3.52	11.14	7.04		
Lower confidence interval	−5.07	−4.27	−2.21	−5.60		
* p*-value, boys vs girls	0.923	0.736	0.036	0.822		
Effect size	0.02	−0.04	0.27	0.04		
